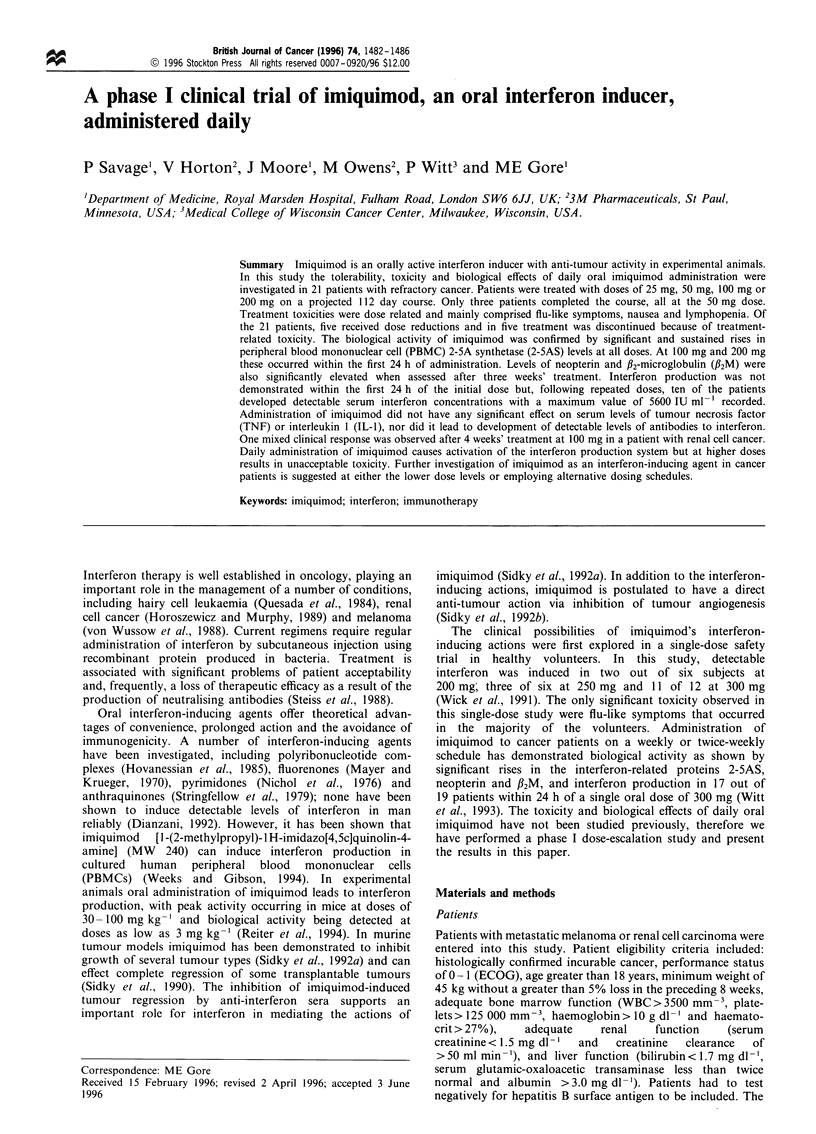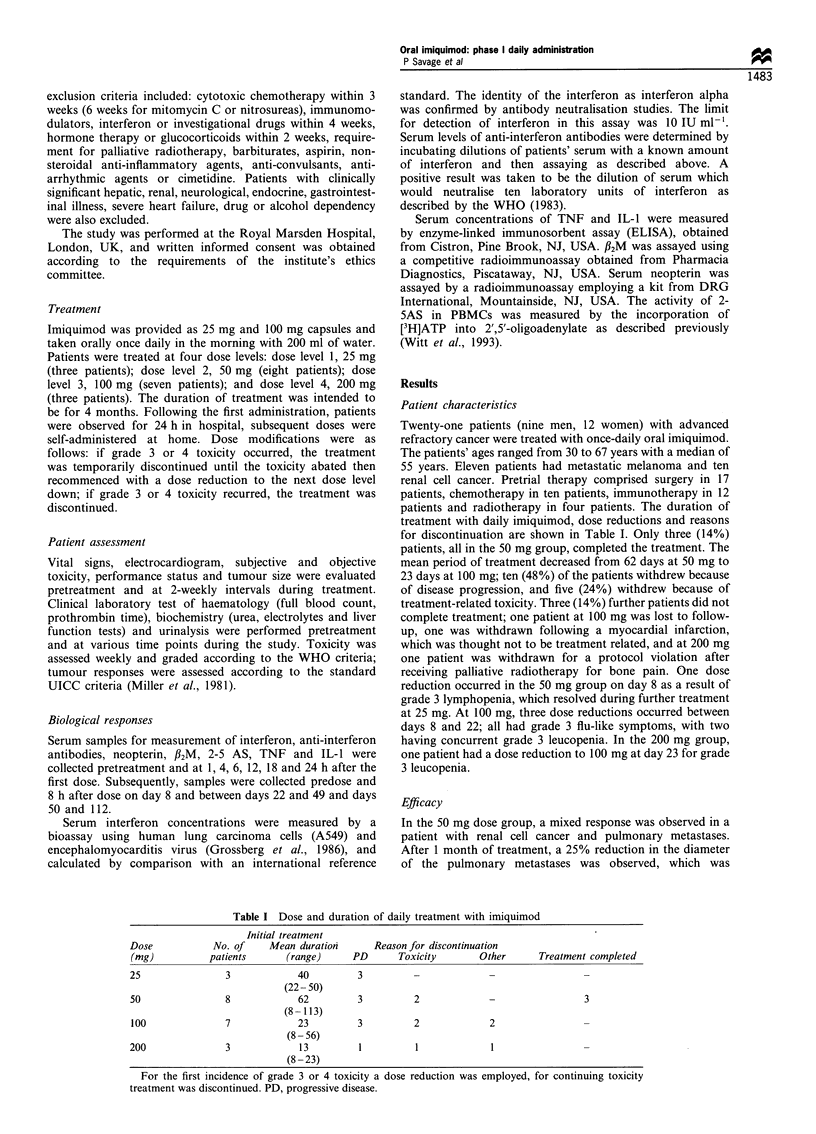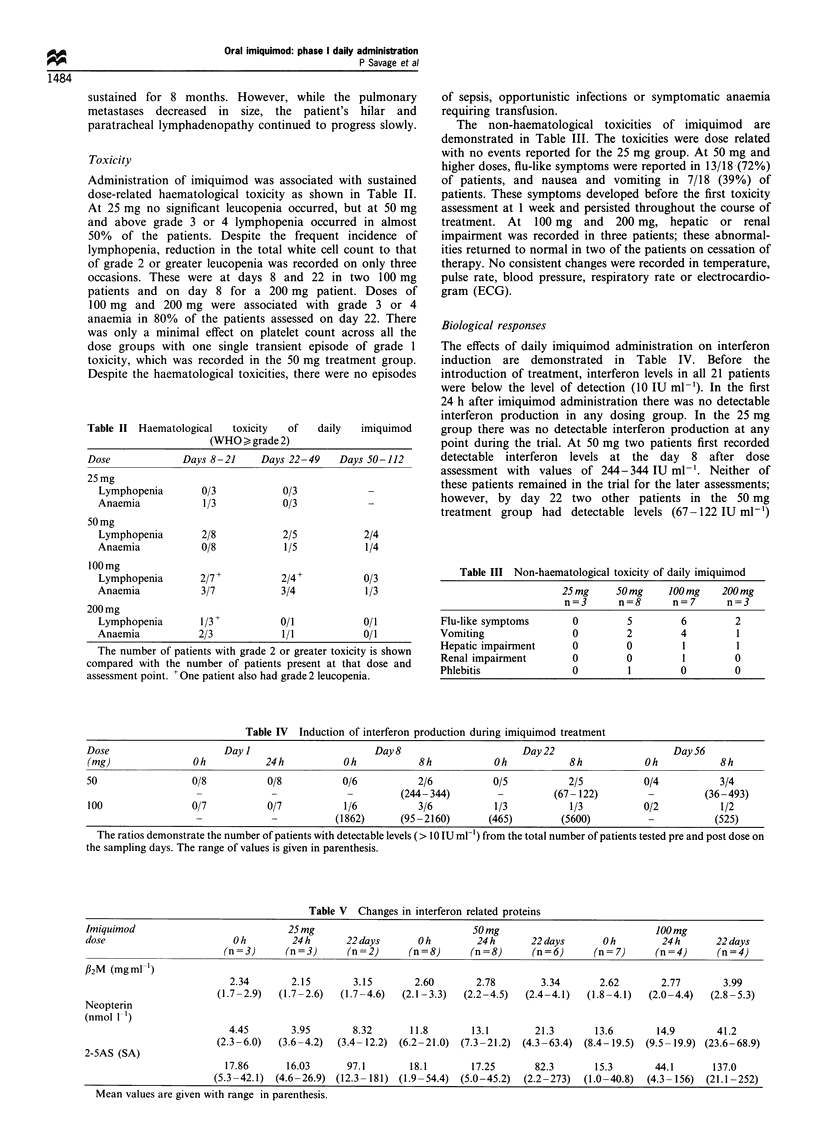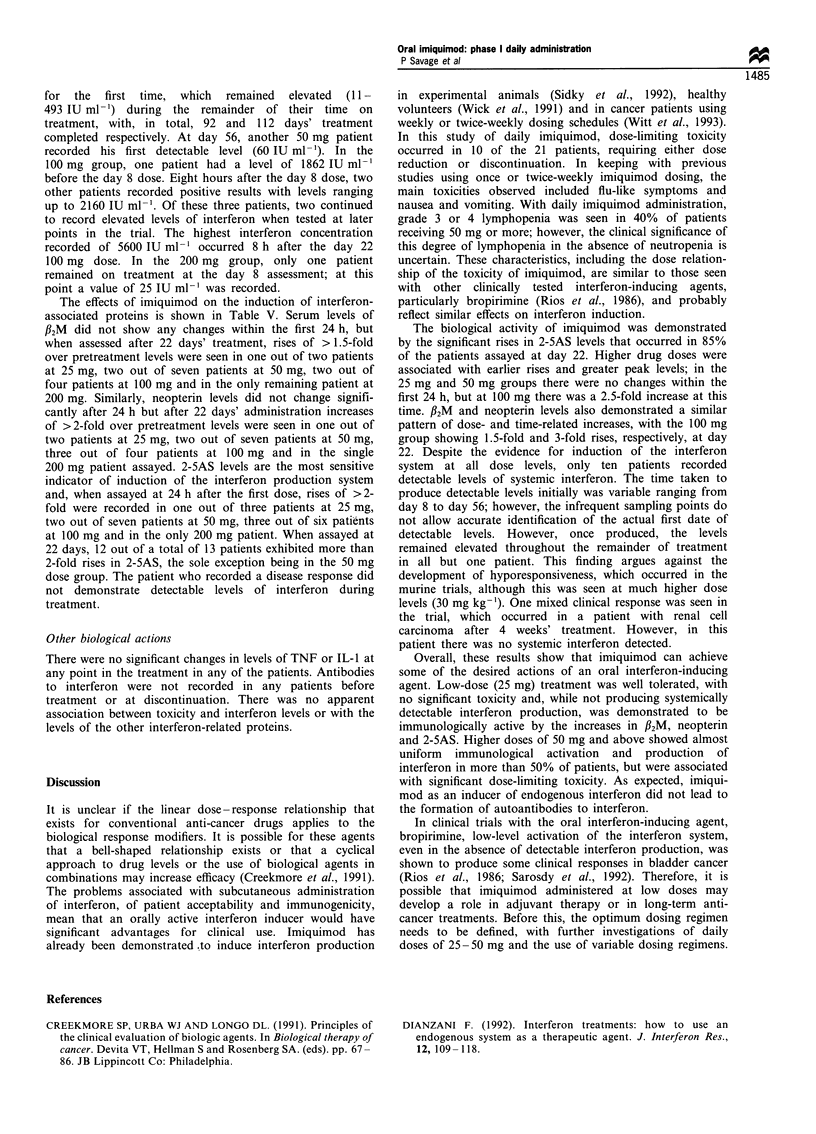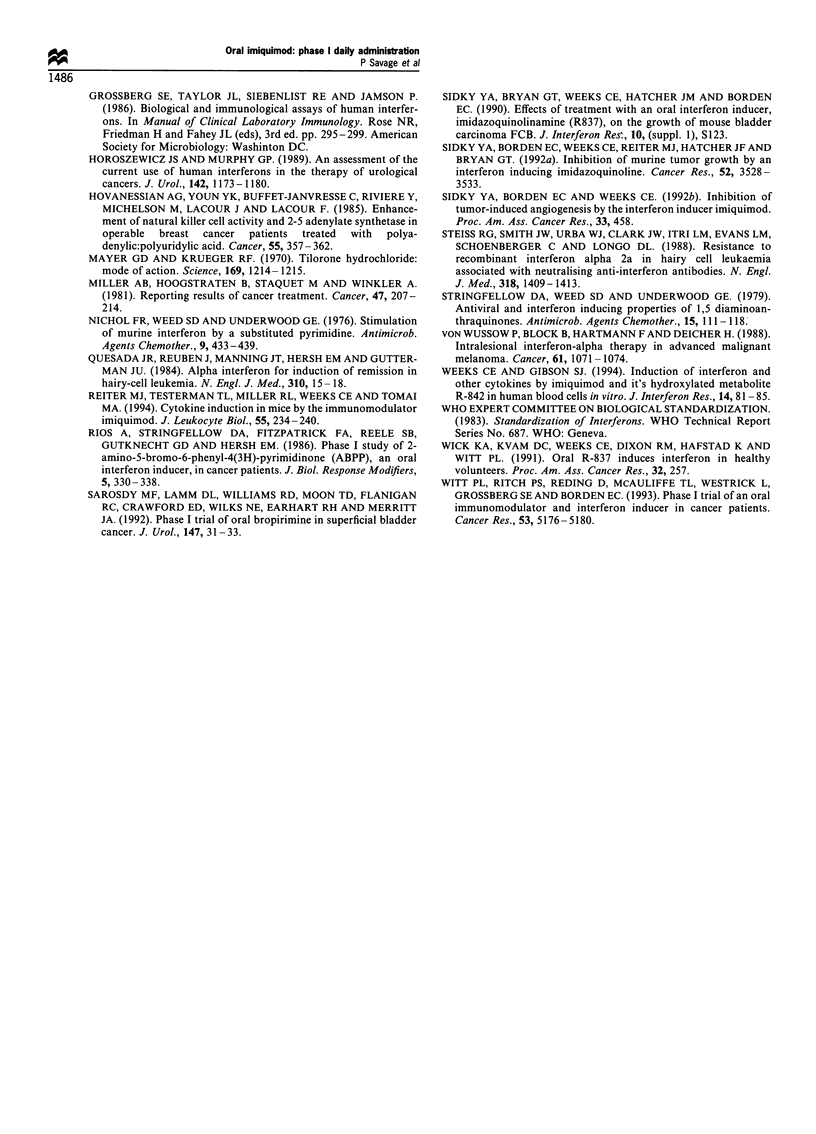# A phase I clinical trial of imiquimod, an oral interferon inducer, administered daily.

**DOI:** 10.1038/bjc.1996.569

**Published:** 1996-11

**Authors:** P. Savage, V. Horton, J. Moore, M. Owens, P. Witt, M. E. Gore

**Affiliations:** Department of Medicine, Royal Marsden Hospital, London, UK.

## Abstract

Imiquimod is an orally active interferon inducer with anti-tumour activity in experimental animals. In this study the tolerability, toxicity and biological effects of daily oral imiquimod administration were investigated in 21 patients with refractory cancer. Patients were treated with doses of 25 mg, 50 mg, 100 mg or 200 mg on a projected 112 day course. Only three patients completed the course, all at the 50 mg dose. Treatment toxicities were dose related and mainly comprised flu-like symptoms, nausea and lymphopenia. Of the 21 patients, five received dose reductions and in five treatment was discontinued because of treatment-related toxicity. The biological activity of imiquimod was confirmed by significant and sustained rises in peripheral blood mononuclear cell (PBMC) 2-5A synthetase (2-5AS) levels at all doses. At 100 mg and 200 mg these occurred within the first 24 h of administration. Levels of neopterin and beta 2-microglobulin (beta 2M) were also significantly elevated when assessed after three weeks' treatment. Interferon production was not demonstrated within the first 24 h of the initial dose but, following repeated doses, ten of the patients developed detectable serum interferon concentrations with a maximum value of 5600 IU ml-1 recorded. Administration of imiquimod did not have any significant effect on serum levels of tumour necrosis factor (TNF) or interleukin 1 (IL-1), nor did it lead to development of detectable levels of antibodies to interferon. One mixed clinical response was observed after 4 weeks' treatment at 100 mg in a patient with renal cell cancer. Daily administration of imiquimod causes activation of the interferon production system but at higher doses results in unacceptable toxicity. Further investigation of imiquimod as an interferon-inducing agent in cancer patients is suggested at either the lower dose levels or employing alternative dosing schedules.


British Journal of Cancer (1996) 74, 1482-1486
© 1996 Stockton Press All rights reserved 0007-0920/96 $12.00

### A phase I clinical trial of imiquimod, an oral interferon inducer, administered daily

P Savage[1], V Horton[2], J Moore[1], M Owens[2], P Witt[3] and ME Gore[1]

[1]*Department of Medicine, Royal Marsden Hospital, Fulham Road, London SW6 6JJ, UK;* [2]*3M Pharmaceuticals, St Paul, Minnesota, USA;* [3]*Medical College of Wisconsin Cancer Center, Milwaukee, Wisconsin, USA.*

**Summary** Imiquimod is an orally active interferon inducer with anti-tumour activity in experimental animals. In this study the tolerability, toxicity and biological effects of daily oral imiquimod administration were investigated in 21 patients with refractory cancer. Patients were treated with doses of 25 mg, 50 mg, 100 mg or 200 mg on a projected 112 day course. Only three patients completed the course, all at the 50 mg dose. Treatment toxicities were dose related and mainly comprised flu-like symptoms, nausea and lymphopenia. Of the 21 patients, five received dose reductions and in five treatment was discontinued because of treatment-related toxicity. The biological activity of imiquimod was confirmed by significant and sustained rises in peripheral blood mononuclear cell (PBMC) 2-5A synthetase (2-5AS) levels at all doses. At 100 mg and 200 mg these occurred within the first 24 h of administration. Levels of neopterin and $\beta_2$-microglobulin ($\beta_2$M) were also significantly elevated when assessed after three weeks' treatment. Interferon production was not demonstrated within the first 24 h of the initial dose but, following repeated doses, ten of the patients developed detectable serum interferon concentrations with a maximum value of 5600 IU ml$^{-1}$ recorded. Administration of imiquimod did not have any significant effect on serum levels of tumour necrosis factor (TNF) or interleukin 1 (IL-1), nor did it lead to development of detectable levels of antibodies to interferon. One mixed clinical response was observed after 4 weeks' treatment at 100 mg in a patient with renal cell cancer. Daily administration of imiquimod causes activation of the interferon production system but at higher doses results in unacceptable toxicity. Further investigation of imiquimod as an interferon-inducing agent in cancer patients is suggested at either the lower dose levels or employing alternative dosing schedules.

**Keywords:** imiquimod; interferon; immunotherapy

Interferon therapy is well established in oncology, playing an important role in the management of a number of conditions, including hairy cell leukaemia (Quesada *et al.*, 1984), renal cell cancer (Horoszewicz and Murphy, 1989) and melanoma (von Wussow *et al.*, 1988). Current regimens require regular administration of interferon by subcutaneous injection using recombinant protein produced in bacteria. Treatment is associated with significant problems of patient acceptability and, frequently, a loss of therapeutic efficacy as a result of the production of neutralising antibodies (Steiss *et al.*, 1988).

Oral interferon-inducing agents offer theoretical advantages of convenience, prolonged action and the avoidance of immunogenicity. A number of interferon-inducing agents have been investigated, including polyribonucleotide complexes (Hovanessian *et al.*, 1985), fluorenones (Mayer and Krueger, 1970), pyrimidones (Nichol *et al.*, 1976) and anthraquinones (Stringfellow *et al.*, 1979); none have been shown to induce detectable levels of interferon in man reliably (Dianzani, 1992). However, it has been shown that imiquimod [1-(2-methylpropyl)-1H-imidazo[4,5c]quinolin-4-amine] (MW 240) can induce interferon production in cultured human peripheral blood mononuclear cells (PBMCs) (Weeks and Gibson, 1994). In experimental animals oral administration of imiquimod leads to interferon production, with peak activity occurring in mice at doses of 30-100 mg kg$^{-1}$ and biological activity being detected at doses as low as 3 mg kg$^{-1}$ (Reiter *et al.*, 1994). In murine tumour models imiquimod has been demonstrated to inhibit growth of several tumour types (Sidky *et al.*, 1992*a*) and can effect complete regression of some transplantable tumours (Sidky *et al.*, 1990). The inhibition of imiquimod-induced tumour regression by anti-interferon sera supports an important role for interferon in mediating the actions of imiquimod (Sidky *et al.*, 1992*a*). In addition to the interferon-inducing actions, imiquimod is postulated to have a direct anti-tumour action via inhibition of tumour angiogenesis (Sidky *et al.*, 1992*b*).

The clinical possibilities of imiquimod's interferon-inducing actions were first explored in a single-dose safety trial in healthy volunteers. In this study, detectable interferon was induced in two out of six subjects at 200 mg; three of six at 250 mg and 11 of 12 at 300 mg (Wick *et al.*, 1991). The only significant toxicity observed in this single-dose study were flu-like symptoms that occurred in the majority of the volunteers. Administration of imiquimod to cancer patients on a weekly or twice-weekly schedule has demonstrated biological activity as shown by significant rises in the interferon-related proteins 2-5AS, neopterin and $\beta_2$M, and interferon production in 17 out of 19 patients within 24 h of a single oral dose of 300 mg (Witt *et al.*, 1993). The toxicity and biological effects of daily oral imiquimod have not been studied previously, therefore we have performed a phase I dose-escalation study and present the results in this paper.

### Materials and methods

### *Patients*

Patients with metastatic melanoma or renal cell carcinoma were entered into this study. Patient eligibility criteria included: histologically confirmed incurable cancer, performance status of 0-1 (ECOG), age greater than 18 years, minimum weight of 45 kg without a greater than 5% loss in the preceding 8 weeks, adequate bone marrow function (WBC > 3500 mm$^{-3}$, platelets > 125 000 mm$^{-3}$, haemoglobin > 10 g dl$^{-1}$ and haematocrit > 27%), adequate renal function (serum creatinine < 1.5 mg dl$^{-1}$ and creatinine clearance of > 50 ml min$^{-1}$), and liver function (bilirubin < 1.7 mg dl$^{-1}$, serum glutamic-oxaloacetic transaminase less than twice normal and albumin > 3.0 mg dl$^{-1}$). Patients had to test negatively for hepatitis B surface antigen to be included. The

Correspondence: ME Gore
Received 15 February 1996; revised 2 April 1996; accepted 3 June 1996

exclusion criteria included: cytotoxic chemotherapy within 3 weeks (6 weeks for mitomycin C or nitrosureas), immunomodulators, interferon or investigational drugs within 4 weeks, hormone therapy or glucocorticoids within 2 weeks, requirement for palliative radiotherapy, barbiturates, aspirin, non-steroidal anti-inflammatory agents, anti-convulsants, anti-arrhythmic agents or cimetidine. Patients with clinically significant hepatic, renal, neurological, endocrine, gastrointestinal illness, severe heart failure, drug or alcohol dependency were also excluded.

The study was performed at the Royal Marsden Hospital, London, UK, and written informed consent was obtained according to the requirements of the institute's ethics committee.

### *Treatment*

Imiquimod was provided as 25 mg and 100 mg capsules and taken orally once daily in the morning with 200 ml of water. Patients were treated at four dose levels: dose level 1, 25 mg (three patients); dose level 2, 50 mg (eight patients); dose level 3, 100 mg (seven patients); and dose level 4, 200 mg (three patients). The duration of treatment was intended to be for 4 months. Following the first administration, patients were observed for 24 h in hospital, subsequent doses were self-administered at home. Dose modifications were as follows: if grade 3 or 4 toxicity occurred, the treatment was temporarily discontinued until the toxicity abated then recommenced with a dose reduction to the next dose level down; if grade 3 or 4 toxicity recurred, the treatment was discontinued.

### *Patient assessment*

Vital signs, electrocardiogram, subjective and objective toxicity, performance status and tumour size were evaluated pretreatment and at 2-weekly intervals during treatment. Clinical laboratory test of haematology (full blood count, prothrombin time), biochemistry (urea, electrolytes and liver function tests) and urinalysis were performed pretreatment and at various time points during the study. Toxicity was assessed weekly and graded according to the WHO criteria; tumour responses were assessed according to the standard UICC criteria (Miller *et al.*, 1981).

### *Biological responses*

Serum samples for measurement of interferon, anti-interferon antibodies, neopterin, $\beta_2$M, 2-5 AS, TNF and IL-1 were collected pretreatment and at 1, 4, 6, 12, 18 and 24 h after the first dose. Subsequently, samples were collected predose and 8 h after dose on day 8 and between days 22 and 49 and days 50 and 112.

Serum interferon concentrations were measured by a bioassay using human lung carcinoma cells (A549) and encephalomyocarditis virus (Grossberg *et al.*, 1986), and calculated by comparison with an international reference standard. The identity of the interferon as interferon alpha was confirmed by antibody neutralisation studies. The limit for detection of interferon in this assay was 10 IU ml$^{-1}$. Serum levels of anti-interferon antibodies were determined by incubating dilutions of patients' serum with a known amount of interferon and then assaying as described above. A positive result was taken to be the dilution of serum which would neutralise ten laboratory units of interferon as described by the WHO (1983).

Serum concentrations of TNF and IL-1 were measured by enzyme-linked immunosorbent assay (ELISA), obtained from Cistron, Pine Brook, NJ, USA. $\beta_2$M was assayed using a competitive radioimmunoassay obtained from Pharmacia Diagnostics, Piscataway, NJ, USA. Serum neopterin was assayed by a radioimmunoassay employing a kit from DRG International, Mountainside, NJ, USA. The activity of 2-5AS in PBMCs was measured by the incorporation of [$^3$H]ATP into 2′,5′-oligoadenylate as described previously (Witt *et al.*, 1993).

### Results

### *Patient characteristics*

Twenty-one patients (nine men, 12 women) with advanced refractory cancer were treated with once-daily oral imiquimod. The patients' ages ranged from 30 to 67 years with a median of 55 years. Eleven patients had metastatic melanoma and ten renal cell cancer. Pretrial therapy comprised surgery in 17 patients, chemotherapy in ten patients, immunotherapy in 12 patients and radiotherapy in four patients. The duration of treatment with daily imiquimod, dose reductions and reasons for discontinuation are shown in Table I. Only three (14%) patients, all in the 50 mg group, completed the treatment. The mean period of treatment decreased from 62 days at 50 mg to 23 days at 100 mg; ten (48%) of the patients withdrew because of disease progression, and five (24%) withdrew because of treatment-related toxicity. Three (14%) further patients did not complete treatment; one patient at 100 mg was lost to follow-up, one was withdrawn following a myocardial infarction, which was thought not to be treatment related, and at 200 mg one patient was withdrawn for a protocol violation after receiving palliative radiotherapy for bone pain. One dose reduction occurred in the 50 mg group on day 8 as a result of grade 3 lymphopenia, which resolved during further treatment at 25 mg. At 100 mg, three dose reductions occurred between days 8 and 22; all had grade 3 flu-like symptoms, with two having concurrent grade 3 leucopenia. In the 200 mg group, one patient had a dose reduction to 100 mg at day 23 for grade 3 leucopenia.

### *Efficacy*

In the 50 mg dose group, a mixed response was observed in a patient with renal cell cancer and pulmonary metastases. After 1 month of treatment, a 25% reduction in the diameter of the pulmonary metastases was observed, which was

**Table I** Dose and duration of daily treatment with imiquimod

| *Dose (mg)* | *Initial treatment No. of patients* | *Initial treatment Mean duration (range)* | *Reason for discontinuation PD* | *Reason for discontinuation Toxicity* | *Reason for discontinuation Other* | *Treatment completed* |
|---|---|---|---|---|---|---|
| 25 | 3 | 40 (22–50) | 3 | – | – | – |
| 50 | 8 | 62 (8–113) | 3 | 2 | – | 3 |
| 100 | 7 | 23 (8–56) | 3 | 2 | 2 | – |
| 200 | 3 | 13 (8–23) | 1 | 1 | 1 | – |

For the first incidence of grade 3 or 4 toxicity a dose reduction was employed, for continuing toxicity treatment was discontinued. PD, progressive disease.

sustained for 8 months. However, while the pulmonary metastases decreased in size, the patient's hilar and paratracheal lymphadenopathy continued to progress slowly.

*Toxicity*

Administration of imiquimod was associated with sustained dose-related haematological toxicity as shown in Table II. At 25 mg no significant leucopenia occurred, but at 50 mg and above grade 3 or 4 lymphopenia occurred in almost 50% of the patients. Despite the frequent incidence of lymphopenia, reduction in the total white cell count to that of grade 2 or greater leucopenia was recorded on only three occasions. These were at days 8 and 22 in two 100 mg patients and on day 8 for a 200 mg patient. Doses of 100 mg and 200 mg were associated with grade 3 or 4 anaemia in 80% of the patients assessed on day 22. There was only a minimal effect on platelet count across all the dose groups with one single transient episode of grade 1 toxicity, which was recorded in the 50 mg treatment group. Despite the haematological toxicities, there were no episodes of sepsis, opportunistic infections or symptomatic anaemia requiring transfusion.

The non-haematological toxicities of imiquimod are demonstrated in Table III. The toxicities were dose related with no events reported for the 25 mg group. At 50 mg and higher doses, flu-like symptoms were reported in 13/18 (72%) of patients, and nausea and vomiting in 7/18 (39%) of patients. These symptoms developed before the first toxicity assessment at 1 week and persisted throughout the course of treatment. At 100 mg and 200 mg, hepatic or renal impairment was recorded in three patients; these abnormalities returned to normal in two of the patients on cessation of therapy. No consistent changes were recorded in temperature, pulse rate, blood pressure, respiratory rate or electrocardiogram (ECG).

*Biological responses*

The effects of daily imiquimod administration on interferon induction are demonstrated in Table IV. Before the introduction of treatment, interferon levels in all 21 patients were below the level of detection (10 IU $ml^{-1}$). In the first 24 h after imiquimod administration there was no detectable interferon production in any dosing group. In the 25 mg group there was no detectable interferon production at any point during the trial. At 50 mg two patients first recorded detectable interferon levels at the day 8 after dose assessment with values of 244–344 IU $ml^{-1}$. Neither of these patients remained in the trial for the later assessments; however, by day 22 two other patients in the 50 mg treatment group had detectable levels (67–122 IU $ml^{-1}$)

**Table II** Haematological toxicity of daily imiquimod (WHO ⩾ grade 2)

| *Dose* | *Days 8–21* | *Days 22–49* | *Days 50–112* |
|---|---|---|---|
| 25 mg | | | |
| Lymphopenia | 0/3 | 0/3 | – |
| Anaemia | 1/3 | 0/3 | – |
| 50 mg | | | |
| Lymphopenia | 2/8 | 2/5 | 2/4 |
| Anaemia | 0/8 | 1/5 | 1/4 |
| 100 mg | | | |
| Lymphopenia | 2/7+ | 2/4+ | 0/3 |
| Anaemia | 3/7 | 3/4 | 1/3 |
| 200 mg | | | |
| Lymphopenia | 1/3+ | 0/1 | 0/1 |
| Anaemia | 2/3 | 1/1 | 0/1 |

The number of patients with grade 2 or greater toxicity is shown compared with the number of patients present at that dose and assessment point. +One patient also had grade 2 leucopenia.

**Table III** Non-haematological toxicity of daily imiquimod

| | *25 mg* n=*3* | *50 mg* n=*8* | *100 mg* n=*7* | *200 mg* n=*3* |
|---|---|---|---|---|
| Flu-like symptoms | 0 | 5 | 6 | 2 |
| Vomiting | 0 | 2 | 4 | 1 |
| Hepatic impairment | 0 | 0 | 1 | 1 |
| Renal impairment | 0 | 0 | 1 | 0 |
| Phlebitis | 0 | 1 | 0 | 0 |

**Table IV** Induction of interferon production during imiquimod treatment

| *Dose* | *Day 1* | | *Day 8* | | *Day 22* | | *Day 56* | |
|---|---|---|---|---|---|---|---|---|
| *(mg)* | *0 h* | *24 h* | *0 h* | *8 h* | *0 h* | *8 h* | *0 h* | *8 h* |
| 50 | 0/8 | 0/8 | 0/6 | 2/6 | 0/5 | 2/5 | 0/4 | 3/4 |
| | – | – | – | (244–344) | – | (67–122) | – | (36–493) |
| 100 | 0/7 | 0/7 | 1/6 | 3/6 | 1/3 | 1/3 | 0/2 | 1/2 |
| | – | – | (1862) | (95–2160) | (465) | (5600) | – | (525) |

The ratios demonstrate the number of patients with detectable levels (> 10 IU $ml^{-1}$) from the total number of patients tested pre and post dose on the sampling days. The range of values is given in parenthesis.

**Table V** Changes in interferon related proteins

| *Imiquimod dose* | *25 mg* *0 h* (n=*3*) | *25 mg* *24 h* (n=*3*) | *25 mg* *22 days* (n=*2*) | *50 mg* *0 h* (n=*8*) | *50 mg* *24 h* (n=*8*) | *50 mg* *22 days* (n=*6*) | *100 mg* *0 h* (n=*7*) | *100 mg* *24 h* (n=*4*) | *100 mg* *22 days* (n=*4*) |
|---|---|---|---|---|---|---|---|---|---|
| $\beta_2M$ (mg $ml^{-1}$) | | | | | | | | | |
| | 2.34 | 2.15 | 3.15 | 2.60 | 2.78 | 3.34 | 2.62 | 2.77 | 3.99 |
| | (1.7–2.9) | (1.7–2.6) | (1.7–4.6) | (2.1–3.3) | (2.2–4.5) | (2.4–4.1) | (1.8–4.1) | (2.0–4.4) | (2.8–5.3) |
| Neopterin (nmol $l^{-1}$) | | | | | | | | | |
| | 4.45 | 3.95 | 8.32 | 11.8 | 13.1 | 21.3 | 13.6 | 14.9 | 41.2 |
| | (2.3–6.0) | (3.6–4.2) | (3.4–12.2) | (6.2–21.0) | (7.3–21.2) | (4.3–63.4) | (8.4–19.5) | (9.5–19.9) | (23.6–68.9) |
| 2-5AS (SA) | | | | | | | | | |
| | 17.86 | 16.03 | 97.1 | 18.1 | 17.25 | 82.3 | 15.3 | 44.1 | 137.0 |
| | (5.3–42.1) | (4.6–26.9) | (12.3–181) | (1.9–54.4) | (5.0–45.2) | (2.2–273) | (1.0–40.8) | (4.3–156) | (21.1–252) |

Mean values are given with range in parenthesis.

for the first time, which remained elevated (11–493 IU $ml^{-1}$) during the remainder of their time on treatment, with, in total, 92 and 112 days' treatment completed respectively. At day 56, another 50 mg patient recorded his first detectable level (60 IU $ml^{-1}$). In the 100 mg group, one patient had a level of 1862 IU $ml^{-1}$ before the day 8 dose. Eight hours after the day 8 dose, two other patients recorded positive results with levels ranging up to 2160 IU $ml^{-1}$. Of these three patients, two continued to record elevated levels of interferon when tested at later points in the trial. The highest interferon concentration recorded of 5600 IU $ml^{-1}$ occurred 8 h after the day 22 100 mg dose. In the 200 mg group, only one patient remained on treatment at the day 8 assessment; at this point a value of 25 IU $ml^{-1}$ was recorded.

The effects of imiquimod on the induction of interferon-associated proteins is shown in Table V. Serum levels of $\beta_2$M did not show any changes within the first 24 h, but when assessed after 22 days' treatment, rises of >1.5-fold over pretreatment levels were seen in one out of two patients at 25 mg, two out of seven patients at 50 mg, two out of four patients at 100 mg and in the only remaining patient at 200 mg. Similarly, neopterin levels did not change significantly after 24 h but after 22 days' administration increases of >2-fold over pretreatment levels were seen in one out of two patients at 25 mg, two out of seven patients at 50 mg, three out of four patients at 100 mg and in the single 200 mg patient assayed. 2-5AS levels are the most sensitive indicator of induction of the interferon production system and, when assayed at 24 h after the first dose, rises of >2-fold were recorded in one out of three patients at 25 mg, two out of seven patients at 50 mg, three out of six patients at 100 mg and in the only 200 mg patient. When assayed at 22 days, 12 out of a total of 13 patients exhibited more than 2-fold rises in 2-5AS, the sole exception being in the 50 mg dose group. The patient who recorded a disease response did not demonstrate detectable levels of interferon during treatment.

### *Other biological actions*

There were no significant changes in levels of TNF or IL-1 at any point in the treatment in any of the patients. Antibodies to interferon were not recorded in any patients before treatment or at discontinuation. There was no apparent association between toxicity and interferon levels or with the levels of the other interferon-related proteins.

### Discussion

It is unclear if the linear dose–response relationship that exists for conventional anti-cancer drugs applies to the biological response modifiers. It is possible for these agents that a bell-shaped relationship exists or that a cyclical approach to drug levels or the use of biological agents in combinations may increase efficacy (Creekmore *et al.*, 1991). The problems associated with subcutaneous administration of interferon, of patient acceptability and immunogenicity, mean that an orally active interferon inducer would have significant advantages for clinical use. Imiquimod has already been demonstrated to induce interferon production in experimental animals (Sidky *et al.*, 1992), healthy volunteers (Wick *et al.*, 1991) and in cancer patients using weekly or twice-weekly dosing schedules (Witt *et al.*, 1993). In this study of daily imiquimod, dose-limiting toxicity occurred in 10 of the 21 patients, requiring either dose reduction or discontinuation. In keeping with previous studies using once or twice-weekly imiquimod dosing, the main toxicities observed included flu-like symptoms and nausea and vomiting. With daily imiquimod administration, grade 3 or 4 lymphopenia was seen in 40% of patients receiving 50 mg or more; however, the clinical significance of this degree of lymphopenia in the absence of neutropenia is uncertain. These characteristics, including the dose relationship of the toxicity of imiquimod, are similar to those seen with other clinically tested interferon-inducing agents, particularly bropirimine (Rios *et al.*, 1986), and probably reflect similar effects on interferon induction.

The biological activity of imiquimod was demonstrated by the significant rises in 2-5AS levels that occurred in 85% of the patients assayed at day 22. Higher drug doses were associated with earlier rises and greater peak levels; in the 25 mg and 50 mg groups there were no changes within the first 24 h, but at 100 mg there was a 2.5-fold increase at this time. $\beta_2$M and neopterin levels also demonstrated a similar pattern of dose- and time-related increases, with the 100 mg group showing 1.5-fold and 3-fold rises, respectively, at day 22. Despite the evidence for induction of the interferon system at all dose levels, only ten patients recorded detectable levels of systemic interferon. The time taken to produce detectable levels initially was variable ranging from day 8 to day 56; however, the infrequent sampling points do not allow accurate identification of the actual first date of detectable levels. However, once produced, the levels remained elevated throughout the remainder of treatment in all but one patient. This finding argues against the development of hyporesponsiveness, which occurred in the murine trials, although this was seen at much higher dose levels (30 mg $kg^{-1}$). One mixed clinical response was seen in the trial, which occurred in a patient with renal cell carcinoma after 4 weeks' treatment. However, in this patient there was no systemic interferon detected.

Overall, these results show that imiquimod can achieve some of the desired actions of an oral interferon-inducing agent. Low-dose (25 mg) treatment was well tolerated, with no significant toxicity and, while not producing systemically detectable interferon production, was demonstrated to be immunologically active by the increases in $\beta_2$M, neopterin and 2-5AS. Higher doses of 50 mg and above showed almost uniform immunological activation and production of interferon in more than 50% of patients, but were associated with significant dose-limiting toxicity. As expected, imiquimod as an inducer of endogenous interferon did not lead to the formation of autoantibodies to interferon.

In clinical trials with the oral interferon-inducing agent, bropirimine, low-level activation of the interferon system, even in the absence of detectable interferon production, was shown to produce some clinical responses in bladder cancer (Rios *et al.*, 1986; Sarosdy *et al.*, 1992). Therefore, it is possible that imiquimod administered at low doses may develop a role in adjuvant therapy or in long-term anti-cancer treatments. Before this, the optimum dosing regimen needs to be defined, with further investigations of daily doses of 25–50 mg and the use of variable dosing regimens.

### References

CREEKMORE SP, URBA WJ AND LONGO DL. (1991). Principles of the clinical evaluation of biologic agents. In *Biological therapy of cancer*. Devita VT, Hellman S and Rosenberg SA. (eds). pp. 67–86. JB Lippincott Co: Philadelphia.

DIANZANI F. (1992). Interferon treatments: how to use an endogenous system as a therapeutic agent. *J. Interferon Res.*, **12**, 109–118.

GROSSBERG SE, TAYLOR JL, SIEBENLIST RE AND JAMSON P. (1986). Biological and immunological assays of human interferons. In *Manual of Clinical Laboratory Immunology*. Rose NR, Friedman H and Fahey JL (eds), 3rd ed. pp. 295–299. American Society for Microbiology: Washinton DC.

HOROSZEWICZ JS AND MURPHY GP. (1989). An assessment of the current use of human interferons in the therapy of urological cancers. *J. Urol.*, **142,** 1173–1180.

HOVANESSIAN AG, YOUN YK, BUFFET-JANVRESSE C, RIVIERE Y, MICHELSON M, LACOUR J AND LACOUR F. (1985). Enhancement of natural killer cell activity and 2-5 adenylate synthetase in operable breast cancer patients treated with polyadenylic:polyuridylic acid. *Cancer*, **55,** 357–362.

MAYER GD AND KRUEGER RF. (1970). Tilorone hydrochloride: mode of action. *Science*, **169,** 1214–1215.

MILLER AB, HOOGSTRATEN B, STAQUET M AND WINKLER A. (1981). Reporting results of cancer treatment. *Cancer*, **47,** 207–214.

NICHOL FR, WEED SD AND UNDERWOOD GE. (1976). Stimulation of murine interferon by a substituted pyrimidine. *Antimicrob. Agents Chemother.*, **9,** 433–439.

QUESADA JR, REUBEN J, MANNING JT, HERSH EM AND GUTTERMAN JU. (1984). Alpha interferon for induction of remission in hairy-cell leukemia. *N. Engl. J. Med.*, **310,** 15–18.

REITER MJ, TESTERMAN TL, MILLER RL, WEEKS CE AND TOMAI MA. (1994). Cytokine induction in mice by the immunomodulator imiquimod. *J. Leukocyte Biol.*, **55,** 234–240.

RIOS A, STRINGFELLOW DA, FITZPATRICK FA, REELE SB, GUTKNECHT GD AND HERSH EM. (1986). Phase I study of 2-amino-5-bromo-6-phenyl-4(3H)-pyrimidinone (ABPP), an oral interferon inducer, in cancer patients. *J. Biol. Response Modifiers*, **5,** 330–338.

SAROSDY MF, LAMM DL, WILLIAMS RD, MOON TD, FLANIGAN RC, CRAWFORD ED, WILKS NE, EARHART RH AND MERRITT JA. (1992). Phase I trial of oral bropirimine in superficial bladder cancer. *J. Urol.*, **147,** 31–33.

SIDKY YA, BRYAN GT, WEEKS CE, HATCHER JM AND BORDEN EC. (1990). Effects of treatment with an oral interferon inducer, imidazoquinolinamine (R837), on the growth of mouse bladder carcinoma FCB. *J. Interferon Res.*, **10,** (suppl. 1), S123.

SIDKY YA, BORDEN EC, WEEKS CE, REITER MJ, HATCHER JF AND BRYAN GT. (1992*a*). Inhibition of murine tumor growth by an interferon inducing imidazoquinoline. *Cancer Res.*, **52,** 3528–3533.

SIDKY YA, BORDEN EC AND WEEKS CE. (1992*b*). Inhibition of tumor-induced angiogenesis by the interferon inducer imiquimod. *Proc. Am. Ass. Cancer Res.*, **33,** 458.

STEISS RG, SMITH JW, URBA WJ, CLARK JW, ITRI LM, EVANS LM, SCHOENBERGER C AND LONGO DL. (1988). Resistance to recombinant interferon alpha 2a in hairy cell leukaemia associated with neutralising anti-interferon antibodies. *N. Engl. J. Med.*, **318,** 1409–1413.

STRINGFELLOW DA, WEED SD AND UNDERWOOD GE. (1979). Antiviral and interferon inducing properties of 1,5 diaminoanthraquinones. *Antimicrob. Agents Chemother.*, **15,** 111–118.

VON WUSSOW P, BLOCK B, HARTMANN F AND DEICHER H. (1988). Intralesional interferon-alpha therapy in advanced malignant melanoma. *Cancer*, **61,** 1071–1074.

WEEKS CE AND GIBSON SJ. (1994). Induction of interferon and other cytokines by imiquimod and it's hydroxylated metabolite R-842 in human blood cells *in vitro*. *J. Interferon Res.*, **14,** 81–85.

WHO EXPERT COMMITTEE ON BIOLOGICAL STANDARDIZATION. (1983). *Standardization of Interferons*. WHO Technical Report Series No. 687. WHO: Geneva.

WICK KA, KVAM DC, WEEKS CE, DIXON RM, HAFSTAD K AND WITT PL. (1991). Oral R-837 induces interferon in healthy volunteers. *Proc. Am. Ass. Cancer Res.*, **32,** 257.

WITT PL, RITCH PS, REDING D, MCAULIFFE TL, WESTRICK L, GROSSBERG SE AND BORDEN EC. (1993). Phase I trial of an oral immunomodulator and interferon inducer in cancer patients. *Cancer Res.*, **53,** 5176–5180.